# Gluten contamination in commercially available gluten-free products in Jordan: a cross-sectional market surveillance study with public health implications

**DOI:** 10.1017/jns.2026.10120

**Published:** 2026-07-07

**Authors:** Leena Mohammad Ahmad, Nour Amin Elsahoryi, Omar Alhaj, Ola Al-Maseimi, Ahmad Al Athamneh, Fatena F. Afaneh, Hana’a Khalaf, Ayman D. Alsheikh, Qutaibah K. Fraihat

**Affiliations:** 1 Department of Clinical Nutrition and Diets, University of Petra, Amman 11196, Jordan; 2 Department of Nutrition and Food Science, Faculty of Allied Medical Sciences, Al-Balqa Applied University, Al-Salt 19117, Jordan; 3 Department of Medical Laboratory Science, Faculty of Allied Medical Sciences, Zarqa University, Zarqa 13110, Jordan; 4 Food Chemistry Laboratory, Jordan Food and Drug Administration, Amman 11181, Jordan

**Keywords:** Coeliac disease, Food safety surveillance, Gluten contamination, Jordan, Market surveillance, Public health nutrition, R5 ELISA, Regulatory compliance

## Abstract

This cross-sectional based market surveillance study examined the level of gluten contamination in commercially manufactured products labelled ‘gluten-free’ in Jordan and compared the results to the globally recognised 20 mg/kg gluten safety limit for people with coeliac disease and other gluten-related disorders. From August 2022 to November 2024, 182 goods from 25 different food categories were tested in retail locations in Jordan’s main governorates. A validated monoclonal-based antibody-sandwich ELISA was used to determine the levels of gluten concentration, and a 95% CI was used to describe the prevalence of contamination. Overall, 47 of 182 products (25.8%; 95% CI: 19.7–32.6%) exceeded 20 mg/kg gluten. With maximum concentrations of 395.8 mg/kg, the highest non-compliance was found in rice-based goods (66.7%), milk products (50.0), and cookies (42.9). On the other hand, with the fact that several categories had rather small sample sizes, no violations were found in any of them. Jordan had a higher rate of contamination than a number of other places, including Europe (0.5%), India (10.8% of packaged items marketed as gluten-free), and Mexico (17.4%). The findings highlight clinically relevant issues with the quality of gluten-free products and support further monitoring, manufacturing supervision, and regulatory actions to better protect customers who depend on strict gluten avoidance guidelines.

## Graphical abstract

**Figure:**
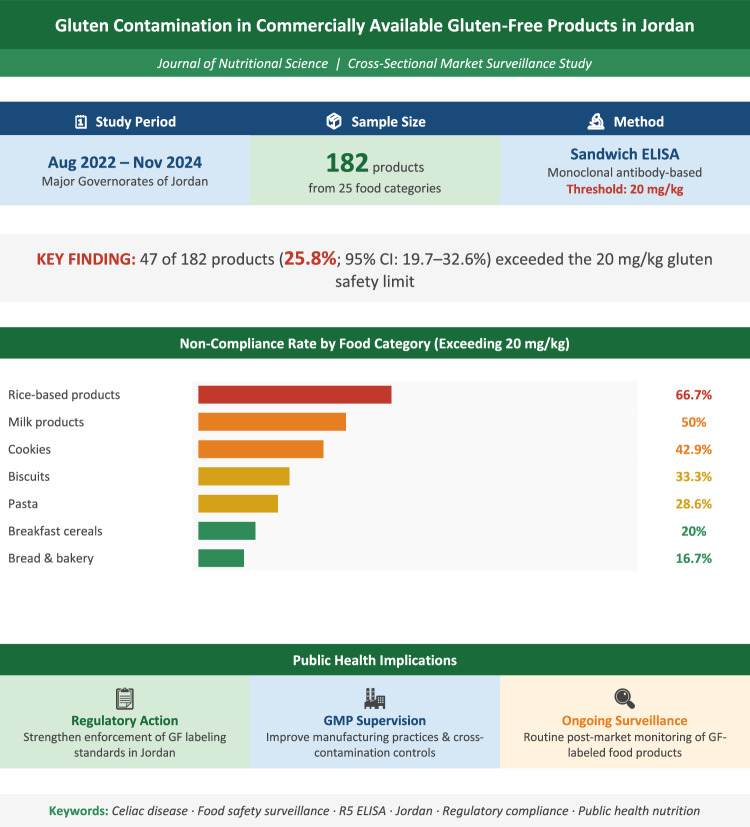

## Introduction

About 0.5–1.26% of people worldwide suffer from coeliac disease, a multisystem autoimmune condition with significant regional differences in prevalence patterns.^(1,2)^ The only proven treatment for this genetically caused enteropathy, which is characterised by gluten-induced villous shrinkage and subsequent malabsorption, is lifetime adherence to strictly gluten-free diets.^(3,4)^

According to recent epidemiological data, the prevalence of coeliac disease is 1–3% in general populations, but it is significantly higher (10–20%) among first-degree relatives of those who have the condition.^(5)^ According to current statistics, incidence is 1.5 times higher in females than in males across a range of populations.^(6)^ The fact that biopsy-confirmed prevalence is 0.7% but global prevalence based on serological markers approaches 1.4% illustrates the importance of histological confirmation for accurate diagnosis.^(7,8)^

Further data from communities in the Middle East and North Africa put doubts on traditional beliefs regarding the geographic distribution of coeliac disease by demonstrating notable prevalence rates comparable to those observed in Western countries.^(9)^ Regional eating habits and a family history of such as higher frequencies of HLA-DQ2 and HLA-DQ8 haplotypes, have an impact on the observed epidemiological patterns showing complex interactions between genetic vulnerability and environmental influences.^(10)^

In Jordan, as in many parts of the Middle East, products labelled ‘gluten-free’ are required to comply with international standards – specifically the Codex Alimentarius states that foods labelled as gluten-free should not have more than 20 ppm of gluten in the food as sold.^(11,12)^ Commission Implementing Regulation (EU) No 828/2014 currently governs information given to consumers in the European Union about the presence or absence of gluten.^(13)^ Based on clinical data showing that intake below this range is protective for the majority of coeliac disease patients, this science-based criterion reflects broad expert consensus.^(14,15)^ Regarding oats, the Codex Alimentarius standard specifies that the permissibility of specially processed oats – those not contaminated with wheat, rye, or barley and containing gluten at levels not exceeding 20 mg/kg – in foods labelled as gluten-free is to be determined at the national level by each adopting country.^(11)^ No formal national-level regulatory determination addressing the inclusion of specially produced oats in gluten-free labelled foods has been established in Jordan. Consequently, oat-derived products were not included in the scope of the present study, and all compliance assessments were conducted exclusively against the universally adopted 20 mg/kg threshold for gluten derived from wheat, rye, and barley. With an overall contamination incidence of 15.12% found in commercially labelled gluten-free products worldwide, systematic evaluations show significant hurdles in maintaining the integrity of gluten-free products.^(16)^ Shared farming equipment, transportation networks, processing plants, and storage settings are examples of cross-contamination mechanisms that may introduce gluten amounts high enough to cause clinical reactions.^(17,18)^ Even though the majority of contamination investigations have been carried out in high-income nations, new data from middle-income environments also points to a risk of contamination that is clinically significant. For instance, Mehtab et al. found that 10.8% of packaged foods labelled as gluten-free in India had gluten levels over the recommended threshold, underscoring the necessity of market surveillance outside of high-income areas.^(19)^ As a middle-income nation in the Middle East, Jordan‘s changing retail food sector lacks thorough data describing gluten contamination. Therefore, the purpose of this study was to produce information pertinent to regulatory monitoring and public health protection by methodically evaluating gluten contamination in items promoted as gluten-free inside Jordan’s retail food system.

## Methods

### Study design and setting

Using a stratified market-based sampling technique, a cross-sectional market surveillance study was carried out throughout Jordan’s retail food industry between August 2022 and November 2024. The study’s methodology was based on established food safety monitoring principles and international best practices for evaluating gluten contamination in order to promote the representation of commercially accessible gluten-free branded foods in the Jordanian market including both products manufactured locally and others imported from various countries around the world.^(20)^

### Sample collection and preparation

Based on an anticipated contamination prevalence of 15% derived from global meta-analyses,^(16)^ with a 95% confidence level and 5% precision, a minimum of 196 products were required for the sample size calculation. A total of 182 products were analysed, representing 93% of the planned sample size and providing enough statistical power (β = 0.80) to detect notable differences in contamination rates across product categories.

The sampling sites included traditional bakeries, individual grocery stores, large supermarket chains, and vendors of specialist health foods from several governorates, including Amman, Irbid, and Zarqa. Online businesses were excluded from the study period. Products were kept in compliance with manufacturer instructions before analysis. label was used to record the brand name, ingredient list, food category, expiration date, and manufacturing information, including the country of manufacture if available, for each sampled product. Product categories, such as cookies, biscuits, rice-based products, seasoning products, milk products, nuts, and nutrition/snack bars, were designated based on the advertised item’s primary form and intended usage.

In order to prepare the sample, solid and semi-solid goods were homogenised using a home blender until a fine, homogenous powder was produced, with about 75% of the material going through a 20-mesh stainless steel screen. Before subsampling, liquid items were well mixed. After homogenisation, representative 5 g subsamples were collected for gluten extraction and analysis. Sample-contact surfaces and blender components were cleaned with alkaline enzyme washing detergent, rinsed with 70% ethanol, and dried between samples to minimise carryover contamination.

### Analytical methods

Gluten quantification was performed at the Food Chemistry Laboratory, Clinical Nutrition Department, University of Petra, using a validated double-antibody-sandwich ELISA (SENSISpec INgezim GLUTEN R5®, Madrid, Spain, version 3.0, 2023). This assay uses R5 monoclonal antibodies with demonstrated specificity for the QQPFP amino acid sequence found in potentially toxic prolamin epitopes from wheat, barley, and rye.^(21,22)^ Samples were processed in accordance with the manufacturer’s protocol, and gluten concentrations were determined from the assay calibration curve.

### Method validation

The R5 sandwich ELISA demonstrated the following validated analytical performance parameters: limit of detection, 1.5 mg/kg; limit of quantification, 3.0 mg/kg; recovery, 95–105% for spiked samples at 10, 20, and 50 mg/kg; reproducibility, 8.7%; repeatability, 7.7%; and inter-assay CV, <10%.^(22)^ All samples underwent duplicate analysis, and positive and negative controls were included in each analytical run. Standard curve parameters were verified (*R* ^2^ > 0.995) for each analytical batch to ensure measurement accuracy.

### Statistical analysis

Descriptive statistical analyses were performed using SPSS version 29.0 and R software version 4.3.0. Products were categorised as compliant (≤20 mg/kg) or non-compliant (>20 mg/kg) according to the internationally used regulatory threshold (11). Contamination rates were calculated with 95% CI using Wilson score intervals. Associations between product categories and contamination status were assessed using chi-square tests. Risk ratios with 95% CI were calculated to quantify contamination risk across product categories. Statistical significance was set at *p* < 0.05.

## Results

### Population-level gluten contamination assessment

Among the 182 commercially available products labelled as gluten-free that were analysed and shown in Table 1, 47 products (25.8%; 95% CI: 19.7–32.6%) exceeded the accepted threshold of 20 mg/kg gluten, whereas 135 products (74.2%) were compliant. Contamination patterns differed significantly across product categories (χ^2^ = 45.23, df = 24, *p* < 0.001), with measured gluten concentrations ranging from undetectable levels to 395.8 mg/kg, substantially above the accepted regulatory threshold.^(11)^

**Table 1.** Gluten contamination analysis and risk stratification of gluten-free labelled products in Jordan

Product category	Product type	Products analysed (*n*)	Gluten range (mg/kg)	Violations > 20 mg/kg (*n*)	Violation rate (%)	95% CI	Risk classification
Critical risk (>40%)							
	Cookies	28	0–395.8	12	42.9	25.4–62.1	Critical
	Rice products	6	0–176.7	4	66.7	30.0–90.3	Critical
	Milk products	2	0–109.8	1	50.0	9.5–90.5	Critical
High risk (25–40%)							
	Seasoning	10	0–141.7	4	40.0	16.8–68.7	High
	Bread/bread mix	11	0–161.4	4	36.4	15.2–64.6	High
	Pastries	8	0–164.8	2	25.0	7.1–59.1	High
Moderate risk (15–25%)							
	Flour/flour mix	13	0–130.5	3	23.1	8.2–50.3	Moderate
	Chocolate	21	0–286.6	4	19.0	7.7–38.9	Moderate
	Biscuits	11	0–54.8	2	18.2	5.1–47.7	Moderate
Low risk (10–15%)							
	Cereals	16	0–128.5	2	12.5	3.5–36.0	Low
	Pasta	25	0–11.3	3	12.0	4.1–29.7	Low
	Cake/cake mix	9	0–374.9	1	11.1	2.0–43.5	Low
Safe products (0%)							
	Potato chips	5	0	0	0	0–43.4	Safe
	Nutrition bars	3	0	0	0	0–56.1	Safe
	Processed meat	2	0	0	0	0–65.8	Safe
	Candy fruits	3	0–5.9	0	0	0–56.1	Safe
	Nuts	1	0	0	0	0–79.4	Safe
	Coffee	1	0	0	0	0–79.4	Safe
	Marshmallow	2	0	0	0	0–65.8	Safe
Single-sample products							
	Egg replacer	1	100.9	1	100.0	20.6–100.0	Requires expanded assessment
	Ketchup	1	148.9	1	100.0	20.6–100.0	Requires expanded assessment
	Chia seeds	1	113.3	1	100.0	20.6–100.0	Requires expanded assessment
	Hazelnut spread	1	176.8	1	100.0	20.6–100.0	Requires expanded assessment
	Weight loss tablets	1	145.2	1	100.0	20.6–100.0	Requires expanded assessment
Total	**All Categories**	**182**	**0–395.8**	**47**	**25.8**	**19.7–32.6**	**Overall Assessment**

### Risk stratification analysis

Category-specific contamination rates are summarised in Table 2. The highest non-compliance rates were observed in rice-based products (66.7%), milk products (50.0%), and cookies (42.9%). Additional elevated rates were observed in seasoning products (40.0%), bread and bread mixes (36.4%), and pastries (25.0%). Intermediate non-compliance was observed in flour and flour mixes, chocolate products, biscuits, cereals, pasta, and cake and cake mixes. No violations were observed in potato chips, healthy nutrition bars, processed meats, crispy candy fruits, nuts, coffee, or marshmallows; however, several of these categories were represented by small sample sizes and should therefore be interpreted cautiously.

**Table 2.** Public health risk assessment and management recommendations by product category

Risk category	Products	Public health priority	Immediate actions	Suggested surveillance frequency	Regulatory priority
Critical (>40%)	Cookies, Rice products, Milk products	Highest concern	Targeted regulatory review	Weekly testing	Highest
High (25–40%)	Seasoning, Bread, Pastries	High concern – Enhanced monitoring needed	Enhanced quality control	Enhanced monitoring	High
Moderate (15–25%)	Flour, Chocolate, Biscuits	Moderate concern – Regular surveillance	Increased surveillance	Monthly testing	Medium
Low (10–15%)	Cereals, Pasta, Cakes	Low concern – Routine monitoring	Routine monitoring	Quarterly testing	Low
Safe (0%)	Potato chips, Healthy nutrition bars, Processed meat, etc.	No violations observed in sampled products	Best practice benchmarks	Annual testing	Maintenance

*Note:* Special consideration required for paediatric coeliac patients who are particularly exposed to contamination effects on growth and development.

### International comparative context

Jordan’s observed non-compliance rate of 25.8% was higher than that reported in several other settings included in Table 3. For direct comparison, emphasis was placed on studies evaluating commercially labelled gluten-free products rather than naturally gluten-free foods. Using this approach, the contamination rate observed in Jordan exceeded those reported in India (10.8%), Mexico (17.4%), Lebanon (19.0%), Europe (0.5%), and Australia (2.7%).

**Table 3.** International comparison of gluten contamination rates in labelled gluten-free (GF) products

Country/region	Study year	Product types	Sample size	Violation rate (%)	Reference
**High-income countries**					
Canada	2008	Cereal-based foods	148	15.0	Gélinas et al.^(28)^
Europe (Italy, Spain, Germany, Norway)	2013	Commercial GF products	205	0.5	Gibert et al.^(23)^
United States	2015	GF food products	275	1.1	Sharma et al.^(29)^
Australia	2018	Manufactured GF foods	256	2.7*	Halmos et al.^(24)^
**Middle-income countries**					
Brazil	2017	GF bakery products	130	21.5	Farage et al.^(30)^
Lebanon	2017	GF-labelled products	173	19.0	Hassan et al.^(25)^
Turkey	2019	Manufactured GF foods	200	17.5	Atasoy et al.^(31)^
India	2021	Labelled gluten-free packaged foods	360	10.8	Mehtab et al.^(19)^
Mexico	2021	GF-labelled foodstuffs	263	17.4	Calderón de la Barca et al.^(26)^
Morocco	2022	GF products	84	23.8	Guennouni et al.^(32)^
**Jordan**	2022	GF-labelled products	182	25.8	**Current study**
China	2025	GF food products	119	13.4	Li et al.^(33)^

*Note:* *2.7% exceeded the Australian gluten-free standard of no detectable gluten (<5 ppm); however, only 0.78% of products analysed were greater than the 20 ppm international standard.

## Discussion

In this market surveillance study of commercially available products labelled as gluten-free in Jordan, 25.8% of sampled products exceeded the accepted threshold of 20 mg/kg gluten.^(11)^ The highest non-compliance rates were observed in rice-based products, milk products, and cookies, and the maximum measured concentration reached 395.8 mg/kg. These findings indicate that a substantial proportion of products marketed as gluten-free in Jordan may expose consumers to clinically meaningful gluten contamination.

The contamination frequency observed in the present study was higher than that reported in several other settings included in our comparison table, including Europe, Australia, Lebanon, Mexico, and India.^(19,23–26)^ This pattern may reflect differences in manufacturing controls, raw material sourcing, supply-chain segregation, regulatory oversight, and market maturity across settings. However, due to variations in sampling contexts, product categories, analytical procedures, and market structures, direct comparisons between research should be regarded with caution.

Since rice is naturally gluten-free, the unusually high contamination found in rice-based goods is noteworthy. According to this research, cross-contact during processing, storage, transportation, or packaging may cause contamination instead of the raw item itself.^(16–18)^ In categories including spice products, flour blends, and bakery-type goods, where ingredient complexity and shared production settings may raise the risk of unintentional gluten exposure, similar mechanisms may also lead to contamination.

These findings are significant from a clinical and public health standpoint because correct gluten-free labelling is essential to the long-term care of coeliac disease.^(3,4)^ In populations with limited access to low-risk product alternatives or specialised dietary counselling, repeated exposure to contaminated items could negatively impact mucosal repair, symptom control, and dietary adherence.^(5,14,15)^ These difficulties may also have a detrimental effect on more general patient-centred outcomes, such as the health-related quality of life of individuals with coeliac disease.^(27)^ Therefore, the current findings emphasise the necessity of better manufacturing controls and increased market surveillance in product categories with greater contamination frequencies.

This study has a number of advantages. It uses a validated R5 sandwich ELISA method for gluten quantification, covers a variety of retail food categories, and offers one of the first organised evaluations of gluten contamination in commercially labelled gluten-free items in Jordan.^(21,22)^ However, a number of restrictions should be taken into account. Sample sizes were uneven across categories, some categories were represented by only a small number of products, and sampling was conducted at a single market level rather than through repeated lot-based testing over time. In addition, product origin was not analysed in sufficient detail to support robust comparisons between locally manufactured and imported foods, and the presence of oat-specific cross-contact risk was not systematically evaluated. These issues should be addressed in future surveillance studies.

Larger numbers within specific product categories, repeated batch-level sampling, and more thorough subgroup analysis by retail channel, ingredient profile, and manufacturing nation should all be included in future studies. To promote the creation of context-specific regulatory and quality assurance measures for gluten-free goods in Jordan and similar regional markets, more work is also required.

## Conclusion

The present findings indicate that a substantial proportion of commercially labelled gluten-free products in Jordan exceed the accepted threshold for safe gluten-free claims. Higher contamination frequencies were observed in selected product categories, particularly rice-based products, milk products, and cookies, highlighting the need for strengthened surveillance and improved manufacturing controls. These data provide an initial evidence base for enhancing regulatory oversight, supporting consumer protection, and guiding future gluten-free market surveillance in Jordan and the broader Middle Eastern region.
